# The effect of Folic Acid, B12, D, and E Vitamins and Melatonin levels in the follicular fluid taken by the Intracytoplasmic Sperm Injection method on pregnancy

**DOI:** 10.12669/pjms.40.3.7929

**Published:** 2024

**Authors:** Kenan Demir, Suleyman Tumer Caliskan, Sebahattin Celik, Muhterem Akdeniz, Rumeysa Yilmaz Goc

**Affiliations:** 1 Kenan Demir Department of Histology and Embryology, Samsun Training and Research Hospital, Samsun University, Samsun, Turkey; 2 Suleyman Tumer Caliskan Department of Urology, Samsun Gazi State Hospital, Samsun, Turkey.; 3 Sebahattin Celik Department of Obstetrics and Gynecology, Balıkesir Private Sevgi Hospital, Balıkesir, Turkey.; 4 Muhterem Akdeniz Department of Obstetrics and Gynecology, In Vitro Fertilization Unit, VM Medical Park Hospital, Samsun, Turkey.; 5 Rumeysa Yilmaz Goc Department of Histology and Embryology Sivas, Sivas Cumhuriyet University, Faculty of Medicine, Turkey

**Keywords:** Antioxidants, In vitro fertilization (IVF), Intracytoplasmic sperm injection (ICSI), Follicular fluid

## Abstract

**Objective:**

It has been demonstrated that the composition of the follicular fluid and many internal/external factors affect success in subfertile couples undergoing intracytoplasmic sperm injection (ICSI). We examined the effect of folic acid, B12, D, and E vitamins and melatonin values in follicular fluid on pregnancy in women with low, normal, and high ovarian reserves who underwent ICSI.

**Methods:**

Our study was conducted at Samsun Medical Park Hospital between January 2021 and February 2022. Follicular fluid induction samples were taken from 96 infertile women with low, normal, and high ovarian reserve, and ICSI was applied. Folic acid, B12, D, and E vitamins and melatonin levels were measured in follicular fluid samples by ELISA method. Statistical analyzes were done with SPSS, and ROC curve analyses were used.

**Results:**

Nine people with poor reserve, 19 people with normal reserve and 14 people with high reserve became pregnant. Folic acid, Vitamin-D, B12, E and melatonin levels were lower in those with poor ovarian reserve than in those with normal and high ovarian reserve (p<0.05). According to the pregnancy test, the probability of pregnancy was 43,783 times higher with high levels of folic acid, while it was 8,096 times higher for vitamin D. While vitamin B12 levels were 31,474 times more likely to be pregnancies, vitamin E levels were 35,227 times higher. For melatonin, the values showed that the probability of pregnancy increased by 11,564 times.

**Conclusions:**

High antioxidants may increase the likelihood of conception in infertile women undergoing ICSI. Therefore, couples who will be treated should be advised to increase these markers, especially melatonin in the follicular fluid.

## INTRODUCTION

Epidemiological studies have indicated that numerous external and internal factors affect the success of IVF/ICSI treatment. The content of follicular fluid represents some of these factors. Furthermore, the content of follicular fluid is correlated with oocyte quality, and its analysis allows us to understand the peri-oocyte cellular complex, which systematically influences the microenvironment of the oocyte.^1^

Many critical cellular pathways in reproduction are folate-dependent, including DNA and RNA synthesis and protein methylation.^2^ Insufficient folic acid dietary intake can lead to megaloblastic anemia and neural tube defects during pregnancy. Daily intake of folic acid tablets beginning several months before conception helps prevent these neural tube defects and some cardiac defects.^3^ Vitamin-D regulates the metabolism of calcium and phosphate.^4^ Data also show the importance of Vitamin-D for fertility, pregnancy outcomes, and breastfeeding. In addition, women with lower serum Vitamin-D levels have been shown to have lower IVF results.^5^

Vitamin B12 is essential for DNA synthesis and methylation and generates cellular energy.^6^ Vitamin B12 deficiency can lead to a broad spectrum of disorders, from fatigue and paresthesia to severe conditions such as pancytopenia and spinal cord degeneration.^7^ Vitamin B12 has been assumed to influence placentation and fetal growth.^6^ Vitamin E has antioxidant properties that reduce oxidative stress. Increased oxidative stress during pregnancy has been associated with preeclampsia, intrauterine growth retardation, and premature rupture of the membrane.^8^

Melatonin detoxifies free oxygen radicals and related oxygen species in addition to mediating G-protein-bound receptors on the cellular membrane.^9^ Melatonin can control seasonal events such as energy metabolism, reproduction, thermogenesis, immune response, body weight control, and growth.^10^ Maternal melatonin is responsible for the fetal circadian rhythm. Likewise, the transfer of the seasonal timing from the mother to the fetus occurs, which prepares the neuroendocrine system for the future environment.^11^

In literature review, folic acid, B12, D, and E vitamins and melatonin were found to have a strong effect on follicular fluid and affect oocyte quality.^5^,^6^,^8^,^11^ However, there has been no study examining the contribution of these vitamins and hormones to pregnancy in women with different ovarian reserve ratios. Therefore, in our study, the effects of folic acid, vitamins B12, D, E and melatonin levels in the follicular fluid on the pregnancy outcomes of infertile women with low, normal and high ovarian reserve who underwent ICSI were evaluated.

## METHODS

This research was carried out at Samsun Medical Park Hospital between January 2021 and February 2022, with the approval of the local ethics committee, numbered 28.04.2020 KAEK2020/3/7. Folic acid, Vitamin-D, B12, E, and melatonin levels were measured using follicular fluid samples from ninety-six infertile patients treated with ICSI cycles. If these values were outside the normal limits, they were excluded from the study. None of the subjects included in the study had a chronic disease. Patients with uterine abnormalities, endometriosis, severe male factors, and a history of uterine surgery were excluded from the study. All subjects gave their informed consent. All subjects were subdivided into low, normal, and high ovarian reserves in response to ovarian stimulation.

An abnormal ovarian reserve test is defined as a low ovarian reserve (i.e., antral follicle count < 7 follicles or AMH < 1 ng/mL) according to the ‘Bologna criteria’^9^ For this study, we took the consensus of the ‘Chinese Medical Association Reproductive Medicine Study Groups Association as standard.^11^ Normal ovarian response was considered serum Basal follicular stimulating hormone (bFSH) < 10 IU/mL and 1 ng/mL < serum AMH < 3.5 ng/mL. High ovarian response was defined as more than 15 oocytes retrieved with controlled ovarian hyperstimulation or a follicle count (>12 to 14 mm) greater than 20. It was proven by transvaginal ultrasound 35-40 days after embryo transfer (ET) those 96 patients responded to ICSI treatment. Clinical pregnancy was defined as the presence of a fetal heartbeat and the appearance of a gestational sac on transvaginal ultrasound.

### Biochemical Analysis:

Follicular fluids samples were collected by the oocyte pickup procedure (OPU) technique; Measurement of folic acid (Sun-Red Bio Company, Cat No. 201-12-1510, Shanghai, China), 25OH Vitamin-D (DIAsource Immuno Assays SA, KAP1917 / F1, Louvain-la Neuve, Belgium), vitamin B12 (Sun-Red Bio Company, Cat No. 201-12-1545, Shanghai, China, vitamin E (Sun-Red Bio Company, Cat No. 201-12-1548, Shanghai, China), and melatonin (Sun-Red Bio Company, Cat No. 201-12-1014, Shanghai, China) concentrations was performed using a commercially available enzyme-linked immunosorbent assay kit (ELISA).

### Statistical Analysis:

Statistical analysis was performed using IBM SPSS Statistics v22 (IBM SPSS, USA). Kolmogorov-Smirnov and Shapiro-Wilk tests were performed to test the normality distribution of the data. A one-way ANOVA test was achieved when the three groups were compared. Kruskal-Wallis test and Mann-Whitney U test were used to evaluate the data that were not normally distributed. Screening tests (sensitivity, specificity, PPV, NPV) and ROC curve analyses were performed to determine folic acid, vitamins D, E, B12, and melatonin cut-off values and reveal pregnancy results.

## RESULTS

The mean age of the subjects was 32.1±5.2 years (range: 19-44). The duration of infertility varied between 1-24 years and was 10.9±5.2 years on normal. Of the cases, 31% (n=30) had a poor reserve, 37% (n=35) had a normal reserve, and 32% (n=31) had a high account (Table-I).

**Table-I:** Selected clinical features of females having normal, high and poor ovarian reserves.

	Normal reserve (n=36)	High reserve (n=31)	Poor reserve (n=30)
Age (y)	31.5 ± 5.2^a^	31.3 ± 4.1^a^	33.9 ± 6.0^a^
BMI (kg/m2)	24.7 (20.1-37.2)^a^	24.1 (20.1-33.1)^a^	24.1 (20.1-32.1)^a^
Duration of infertility (y)	10.6 ± 4.6^a^	9.7 ± 4.2^a^	12.4 ± 6.2^a^

*Data are presented as mean ± SD or median (min-max) as appropriate and ANOVA and Kruskal-Wallis tests. Every superscript letter refers to females having normal, high, and poor ovarian reserves in whose column values there is no significant difference between each other at the p<0.05.

Pregnancy results of a total of 96 patients were positive in 42 (43.8%) and negative in 54 (56.2%). Clinical pregnancy was proven in nine people with poor reserves, 19 people with normal reserves, and 14 people with high reserves.

Based on the significance of these parameters in the context of ovarian reserve and pregnancy, follicular fluid samples collected during the oocyte retrieval procedure and ROC analyses to determine their predictive values and potential for use as a diagnostic test are shown in Table-II.

**Table-II:** Results of the ROC curve analysis and cut-off, sensitivity, and specificity values of folic acid, vitamins B12, D, E, and melatonin for predicting pregnancy in the study population.

	Cut-off	Sensitivity	Specificity	Positive predictive value	Negative predictive value	The area under the curve	95% confidence interval	p
Folic acid	≥ 19.02	45.24	100	95	69.73	0.681	0.578-0.773	0.002
Vit. D	≥ 14.61	45.2	98.1	79.16	68.05	0.768	0.671-0.848	0.001
Vit. B12	≥244.48	54.76	98.15	92	73.23	0.742	0.643-0.826	0.001
Vit. E	≥ 0.57	73.8	92.59	88.57	81.96	0.874	0.799-0.949	0.001
Melatonin	≥ 59.86	78.6	75.92	71.73	82	0.822	0.730-0.892	0.001

**Fig.1:**
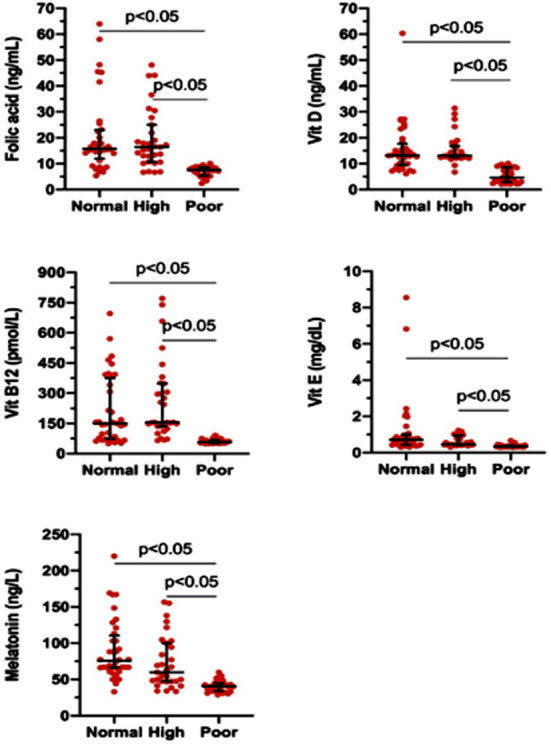
Levels of folic acid, vitamins D, B12, E, and melatonin in the follicular fluid of women having normal, high, and poor ovarian reserves. Data are presented as median with interquartile range. The median levels of folic acid, vitamin-D, B12, E, and melatonin of females with a low ovarian reserve were significantly lower than that of females with normal and high ovarian reverses (p<0.05). No significant differences in folic acid, vitamins D, B12, E, and melatonin were detected between women with normal and high ovarian reserves (p>0.05).

The levels of folic acid, vitamins D, B12, and E, and melatonin in the follicles of women with normal, high, and poor ovarian reserve are shown in Fig.1 Mean folic acid, Vitamin-D, B12, E, and melatonin levels of women with weak ovarian reserve were lower than women with normal and high ovarian reversals (p<0.05). However, the values of folic acid, vitamins D, B12, E, and melatonin were similar between women with normal and high ovarian reserve (p>0.05). According to the pregnancy test, for folic acid 19.02 cut-off value, sensitivity was 45.24%, specificity was 100%, positive predictive value was 95%, negative predictive value was 69.73%, and precision was 75%. The area under the ROC curve was 68.1±6%. Pregnancy probability was 43.783 times higher in cases with folic acid levels of 19.02 and above (95% CI, p=0.000, p<0.01).

Sensitivity was 45.2%, specificity was 98.1%, PPV was 79.16%, NPV was 68.05%, and precision was 70.83% for the cut-off value of 14.61 for vitamin-D measurement. The area under the ROC curve was 76.8%±4.8%. Pregnancy probability was 8.096 times higher in cases with a Vitamin-D level of 14.61 and above (95% CI, p=0.000. p<0,01).

The cut-off value for vitamin B12 measurement was found to be 244.48. The sensitivity for this vitamin B12 value was 54.76%, specificity 98.15%, PPV 92%, NPV 73.23%, and precision 78.12%. The area under the ROC curve was estimated to be 74.2%, and the standard error to be 5.6%. When the vitamin B12 level was 244.48 and above, 31.474 times more pregnancy was seen (95% CI, p=0.000, p<0.01).

Pregnancy was 35.227 times higher for vitamin E, which had a 0.57-cut-off value compared to the pregnancy test (95% CI, p=0.000, p<0.01). In addition, sensitivity was 73.80%, specificity 92.59%, PPV 88.57%, NPV 81.96%, and precision 84.37%. Therefore, the area under the ROC curve was calculated as 87.4%±3.8%. For melatonin measurement with a cut-off value of 59.86, sensitivity was 78.6%, specificity 75.92%, PPV 71.73%, NPV 82%, and accuracy 77.08% (95% CI, p=0.000, p<0.01). These values showed that the probability of pregnancy increased 11.564 times. The area under the ROC curve was 82.2%±4.5%.

**Fig.2:**
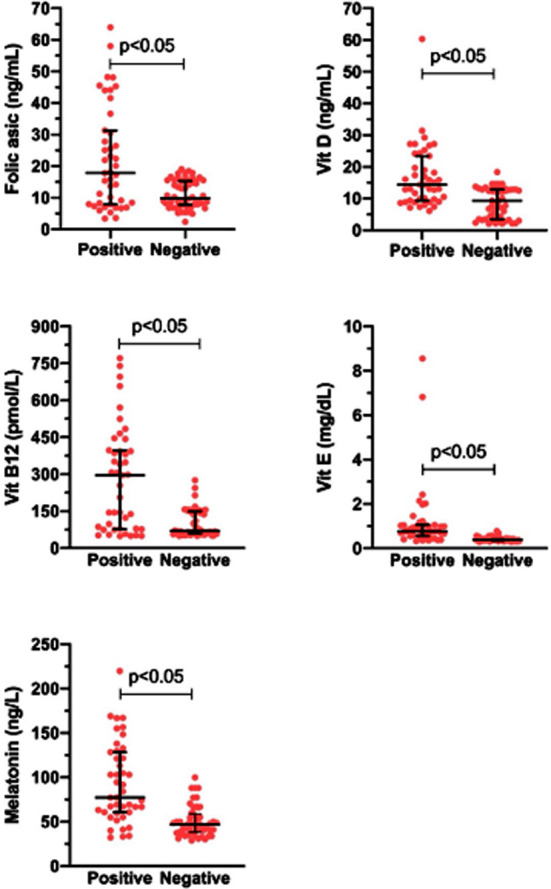
Levels of folic acid, vitamins D, B12, E, and melatonin in the follicular fluid of women with or without pregnancy. Data are presented as median with interquartile range. The median levels of folic acid, vitamins D, B12, E, and melatonin of pregnant women (n: 42) were significantly higher than those of women without pregnancy (n:54) (p<0.05).

## DISCUSSION

This study measured folic acid antioxidants, including folic acid, vitamins B12, D, and E, and melatonin to determine their role in pregnancy outcomes in infertile women with low, normal, and high ovarian reserves undergoing IVF and ICSI procedures. Studies have shown that the effects of different levels of ovarian reserve on infertility are also different. However, its pathways are not known exactly.^12^,^13^ Free radicals result from the leakage of high-energy electrons as they move along the electron carrier chain. Free radicals have many harmful effects, such as DNA damage. In addition, oocytes also produce free oxygen radicals. Bedaiwy et al. revealed an association between increased reactive oxygen species (ROS) during IVF cycles, embryo fragmentation, and decreased fertilization rates.^14^

Folic acid, vitamins D, B12, E, and melatonin used in our study have antioxidant properties. Twigt et al. revealed that a low folate level in the follicular fluid leads to increased inflammatory changes. Furthermore, folate levels affect the follicular microenvironment through intraovarian pathways and peripheral tissue changes. Folate alters follicular metabolism and oocyte maturation via extra-ovarian mechanisms.^1^ Nevertheless, they did not examine follicular fluid and used vitamin E, folic acid, and other antioxidants.^15^ Another study determined optimal antioxidant levels related to positive effects on sperm function tests and assisted reproductive therapy.^16^ Our study proved that high levels of folic acid in follicular fluid are associated with increased pregnancy rates in ICSI.

In the most comprehensive study investigating the relationship between pregnancy rates and vitamin-D levels through IVF/ICSI, serum vitamin-D deficiency has been shown to affect pregnancy after IVF/ICSI treatment.^5^

However, another study showed that vitamin-D positively correlates with the normal fertilization rate.^17^ vitamin-D has been shown to affect implantation and lead to an immunomodulatory effect.^16^,^18^ However, we showed that a high vitamin-D level in follicular fluid was related to increased pregnancy rates in ICSI. Vitamin B12, also known as cobalamin, plays a crucial role in various biological processes, including DNA synthesis, methylation, and cellular energy production.^19^

In a study to compare vitamin B12 levels in follicular fluid with ICSI, patients were given a Mediterranean diet, and its relationship with pregnancy success was investigated. In this study, a Mediterranean diet rich in vitamins (antioxidants) increased pregnancy success in ICSI with live birth.^20^ Our analysis also supported this study. We found that the success of ICSI was positively correlated with the level of vitamin B12 in the follicular fluid.

The relationship between vitamin E and ICSI has yet to be investigated.^21^ Our study examined the level of vitamin E in the follicular fluid and found it directly proportional to pregnancy rates. The relationship between vitamin E and ICSI was primarily examined on the PCOS axis^22^,^23^, and the effects of vitamin E on male infertility were investigated.^1^ However, no studies have investigated vitamin E’s effect on pregnancy rates in ICSI. Therefore, our study examined the level of vitamin E in follicular fluid, and it was found to be directly proportional to pregnancy rates.

Jing Tong et al. found a relationship between melatonin levels in follicular fluid and age, AMH and bFSH, and ovarian reserves. The authors also found a correlation between melatonin levels and IVF outcomes.^11^ Mature oocytes have been shown to have significantly higher melatonin levels than immature eggs.^24^ It has been suggested that melatonin greatly influences ART conception cycles.^25^ Our research findings are in line with the research results mentioned above. In addition, a positive correlation was found between melatonin levels and pregnancy rates.

### Limitations of the study:

Because the study was conducted in a single center, the generalizability of the findings to other settings or populations remains limited, as there may be differences in patient characteristics, protocols, and laboratory techniques at different centers.

## CONCLUSION

This study investigated the role of folic acid, vitamins B12, D, and E, and melatonin in pregnancy outcomes in infertile women undergoing IVF and ICSI procedures. The results showed several positive associations between the levels of these vitamins and hormone in follicular fluid and increased pregnancy rates. Specifically, higher levels of folic acid, Vitamin-D, E, and melatonin were associated with improved outcomes.

Although a single-center study, this adds to the existing literature regarding the potential benefits of folic acid, vitamins D, B12, E, and melatonin in fertility treatments. The findings suggest that optimizing the levels of these vitamins and hormone in the follicular fluid may be a promising approach to increase the success of IVF and ICSI procedures. Further research can guide clinical practice and improve reproductive outcomes for infertile women.

### Authors’ Contribution:

**KD:** Conceived, designed, did statistical analysis & editing of the manuscript and is responsible for the integrity of the research. **STC**, **SC** & **MA:** Did data collection and manuscript writing. **RYG:** Did review and finally approved the manuscript.
